# Neuromuscular Electrical Stimulation in Patients With Severe COVID-19 Associated With Sepsis and Septic Shock

**DOI:** 10.3389/fmed.2022.751636

**Published:** 2022-02-16

**Authors:** Renato Fraga Righetti, Samantha Torres Grams, Wesla Neves da Silva Costa, Leandro Teixeira Saraiva, Isabel Chateaubriand Diniz de Salles, Wellington Pereira Yamaguti

**Affiliations:** Hospital Sírio-Libanês, São Paulo, Brazil

**Keywords:** COVID-19, sepsis, physiotherapy, neuromuscular electrical stimulation, muscle mass

## Abstract

**Background:**

Neuromuscular electrical stimulation (NMES) can be applied to critically ill patients. However, its results on muscle strength and functionality in patients with COVID-19 are unknown.

**Objective:**

Evaluate the effects of intervention with NMES on muscle mass and functionality of patients with severe COVID-19 associated with sepsis and septic shock.

**Methods:**

Seven patients with COVID-19 associated with sepsis or septic shock were selected, but only 5 patients completed all days of the intervention with NMES. The intervention was performed by a single physiotherapist on 7 consecutive days in a daily session of 40 min. The outcome measures were the femoris cross-sectional area; thickness of the anterior compartment of the quadriceps muscle; rectus femoris echogenicity; International Classification of Functioning, Disability, and Health (ICF)-muscle strength; PFIT-s, DEMMI, and the SOMS; feasibility, and safety. The patients were evaluated on days 1, 5, and 8.

**Results:**

The rectus femoris cross-sectional area decreased significantly from days 1 to 8, but showed maintenance of the thickness of the anterior compartment of the quadriceps muscle from days 1 to 8. The MRC score increased significantly from days 1 to 5 and kept this improvement until day 8. All patients showed an increase in the MRC score and reduction of the ICF-muscle strength, meaning improved muscle strength from days 1 to 8. The PFIT-s increased significantly from days 1 to 5 and improved until day 8 compared to day 5. DEMMI and SOMS score increased significantly on day 8 compared to days 1 and 5.

**Conclusion:**

Rehabilitation with NMES showed improvement in muscle strength and functionality of patients in this study with a potential protective effect on muscle mass loss in patients with critical COVID-19 associated with sepsis and septic shock. This study is the first report of the potential effects of neuromuscular electrical stimulation in patients with severe COVID-19 associated with sepsis and septic shock.

## Introduction

The Coronavirus disease 2019 (COVID-19) is caused by novel Severe Acute Respiratory Syndrome Coronavirus 2 (SARS-CoV-2) (1). The virus spread rapidly through the world population and several hospitals have produced guidelines for the respiratory management of these patients (2, 3). Most patients have a mild form of the disease, but 5% of patients present severe lung injury and required intensive care (4). These patients may develop ICU-acquired weakness (5).

Early mobilization in the intensive care unit (ICU) is proven to be effective in preventing muscle atrophy and functional disability. However, it is not necessarily applicable to all patients (6). Therefore, neuromuscular electrical stimulation (NMES) has been used as an additional rehabilitation strategy for critically ill patients (7). Studies using electrical muscle stimulation in septic patients have conflicting results depending on the titred stimulation frequency used. These studies showed that low stimulation frequency electrical stimulation was ineffective to preserve muscle mass (8) and high stimulation frequency electrical stimulation was able to increase strength (9). Carraro et al. (10) suggest that frail persons post-COVID-19 infection with muscle weakness or persons in prolonged inactivity for pandemic-related restriction may benefit from the full-body exercise program associated with NMES. However, these effects are unknown in patients in the acute phase of the disease with severe COVID-19.

The present study aims to describe our clinical protocol in the treatment with neuromuscular electrical stimulation of patients with COVID-19 associated with sepsis and septic shock during their acute intensive care unit stay and to discuss intervention responses in skeletal muscle mass and functional performance.

## Methods

All participants signed the Informed Consent Term, previously approved by the Ethics Committee of the Hospital Sírio-Libanês (number 3,999,139). This case series was conducted at the adult intensive care units of Hospital Sírio-Libanês, São Paulo, Brazil, and all approved ethical protocols were followed.

### Patients

Seven patients with COVID-19 associated with sepsis or septic shock with age ≥ 18 years were selected. Sepsis diagnosis was defined by the Third International Consensus Definitions for Sepsis and Septic Shock (Sepsis-3) (11). Furthermore, patients should have the capacity to walk independently before hospitalization classified by mean of the Expanded Disability Status Score (EDSS) ≤ 6 (12) and immobilization period without walking ≤ 7 days.

### Candidate Patients for Neuromuscular Electrical Stimulation

The inclusion criteria for starting NMES to critically ill patients include body mass index (BMI) ≤ 35 kg/m^2^; without skin lesions, cardiac pacemaker, infection or trauma in lower limbs, neuromuscular diseases, use of neuromuscular blockers, polyneuropathy, and imminent risk of death in less than 48 hours (Simplified Acute Physiology Score III - SAPS III ≤ 80). The exclusion criteria for intervention were infarction and/or need for mechanical cardiopulmonary bypass devices or the need for intra-aortic balloon during ICU hospitalization.

### Clinical Assessment

In the ICU admission, patients were evaluated and classified to clinical severity according to the Simplified Acute Physiology Score III (SAPS III) (13) and the Sequential Organ Failure Assessment (SOFA) (14). In addition, we collected clinical and neurological parameters. SAPS III and SOFA assessments were performed by the medical team of the intensive care unit.

### Outcome Measures

**Figure 1:**
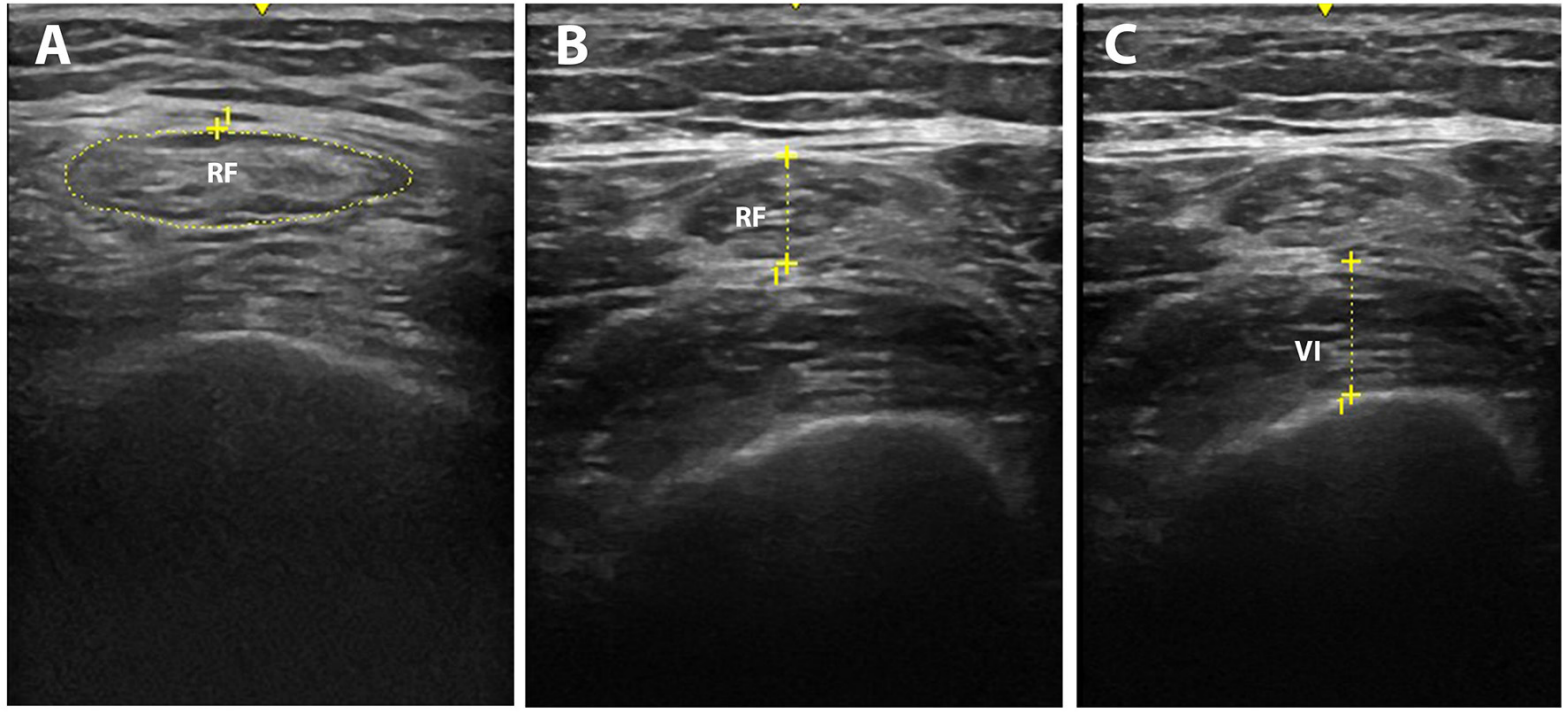
Representative muscle ultrasound image methods: rectus femoris cross-section area **(A)**, the thickness of the anterior compartment of the quadriceps muscle [rectus femoris **(B)** and vastus intermedius **(C)**].

Muscle mass was assessed using ultrasonography. Patients were evaluated concerning rectus femoris cross-sectional area (cm^2^), the thickness of the anterior compartment of the quadriceps muscle (rectus femoris and vastus intermedius) (cm), and rectus femoris echogenicity (pixels) (5). The transducer was positioned perpendicular to the longitudinal axis of the thigh in 80% of the distal distance between the anterosuperior iliac spine and the upper midpoint of the patella to obtain measurements of the rectus femoris cross-sectional area, and thickness of the anterior compartment of the quadriceps muscle (rectus femoris and vastus intermedius). The measurements were performed using B-mode ultrasound (Logiq e ultrasound, GE Healthcare, USA) (Figure 1).

Functionality was assessed by the International Classification of Functioning, Disability, and Health (ICF) using the muscle strength domains (b730) based on the Medical Research Council (MRC) score for global strength (5). The ICF scores used were: 0–58 to 60 (without significant changes); 1–48 to 57 (slight loss); 2–31 to 47 (moderate loss); 3–4 to 30 (severe loss); and 4–0 to 3 (maximum loss). The MRC score is a voluntary method and depends on the understanding and collaboration of the patients. For this reason, in the case of patients on mechanical ventilation, it was evaluated only after interrupting sedation (15). In addition, we assessed functionality by the Physical Function ICU Test-scored (PFIT-s), Morton Mobility Index (DEMMI), and the Surgical Intensive Care Unit Optimal Mobilization Score (SOMS).

PFIT-s examine the capacity of the patient in the sit-to-stand level of assistance; maximal marching on the spot duration and number of steps; and shoulder flexion strength, and knee extension strength. The PFIT-s score ranges from 0 (unable to perform activities) to 10 (high physical functioning) (16). DEMMI is composed of 15 hierarchical mobility activities (bed-based, chair-based, static balance, walking-related, and dynamic balance). The total score is converted with Rasch Analysis with a score range from 0 (poor mobility) to 100 (high levels of independent mobility) (16). PFIT-s and DEMMI depend on the understanding and collaboration of the patients, and it was performed after interrupting the sedation. The SOMS score ranges from 0 (indicating that no mobilization should be considered since deemed to be futile, as for patients in a terminal unstable clinical condition such as those with intracranial hypertension or severe systemic hemodynamic and respiratory insufficiency) to 4 (patients able to ambulate) (17).

The rectus femoris cross-sectional area (cm^2^), the thickness of the anterior compartment of the quadriceps muscle, ICF-muscle strength, PFIT-s, DEMMI, and SOMS were evaluated on days 1, 5, and 8 of start intervention with neuromuscular electrical stimulation. All measurements were performed by the same physiotherapist on days 1, 5, and 8 and was blind to the interventions that were applied to the patients.

### Neuromuscular Electrical Stimulation

The NMES was performed after interrupting the neuromuscular blocker with the patient in the supine position in the ICU bed with 30–60 degrees of the hips and knees joint flexion. The ICU bed itself was used to achieve the positioning of the patient necessary for intervention with NMES. Two pairs of self-adhesive electrodes (size 9 × 5 cm, SPES Medica Brazil Ltda, São Paulo, Brazil) were positioned distally over the motor area of vastus medialis and vastus lateralis muscles, and the other two were placed 5 cm below the inguinal region. The location of the electrodes was marked on the skin with a surgical marking pen to ensure application in the same location on subsequent days. This position of the NMES electrodes is capable of stimulating the motor points of the quadriceps muscles (18).

The parameters used were stimulation frequency of 100 Hz, a stimulation pulse width of 350 μs, a ramp-up time of 1 s, time on of 4 s, ramp-dow of the stimulation of the 1 s, and time off of 12 s. The stimulation pulse width was performed with charge-balanced biphasic pulses and trapezoidal waves. In awake patients, the intensity was established with the maximum muscle contraction tolerated by the patient. In sedated patients, it NMES was adjusted with 50% above the minimum necessary to generate a visible contraction (8). The stimulation frequency was based on Rodriguez et al. (9) that showed that the high stimulation frequency electrical stimulation presented a preventive effect in the progression of muscle weakness in patients having severe sepsis requiring mechanical ventilation. During the intervention with NMES, no voluntary muscle movement was requested.

The treatment with NMES was interrupted if the patient presented cardiorespiratory instability, high fever (above 39°C), development of muscle fatigue, pain above 7 on the Visual Analog Scale (VAS), or pain above 2 on the Pain Assessment in Advanced Dementia (PAINAD) scale (19).

The application of NMES was carried out by the same physiotherapist on 7 consecutive days in a daily session of 40 minutes. For the treatment, we used the NMES device (Neurodyn II; IBRAMED; Amparo; São Paulo; Brazil). The physiotherapist involved in the NMES intervention did not participate in the outcome assessment and was blind to the results.

### Feasibility and Safety

Feasibility was determined based on adherence and safety was evaluated based on the incidence of adverse events. Adverse events were considered: hemodynamic instability, respiratory instability, skin injury, and bruises.

### Statistical Analysis

Data were assessed for normality using the Shapiro-Wilk test. Parametric variables are presented as mean and standard error. Categorical data are presented as the absolute (*n*) and relative frequency (%). Change in the muscle mass and functional capacity was assessed by repeated measure analysis of variance. Statistical significance was indicated by a *P* < 0.05.

## Results

Seven patients attended the NMES sessions. One patient stopped the treatment of NMES and one patient died on day 8 (patients 4 and 6); therefore, data for these patients were not included in the outcomes of all patients; only their data are displayed in the individual patient values graph. The demographic characteristics of the patients are shown in Table 1.

**Table 1:** Characteristics of patients with COVID-19 associated with sepsis and septic shock during ICU and hospital stay.

**Demographic characteristics and clinical characteristics**													
**Patient**	**Age (y)**	**Gender**	**BMI**	**SOFA**	**SAPS III**	**COVID-19 severity**	**Sedation**	**Vasoactive drug**	**Neuromuscular blocker**	**Hydrocortisone**	**IMV days**	**ICU stay**	**Hospital stay**
1	67	Female	28.9	8	67	Critical illness	Yes	Yes	Yes	Yes	6	11	24
2	65	Female	30.2	0	38	Critical illness	Yes	Yes	Yes	Yes	4	15	22
3	72	Male	30.9	7	57	Critical illness	Yes	Yes	Yes	Yes	8	12	27
4	61	Male	31.7	0	46	Critical illness	Yes	Yes	Yes	Yes	6	28	28
5	67	Male	32.6	5	50	Critical illness	No	No	No	Yes	0	9	14
6	75	Male	31.2	7	90	Critical illness	Yes	Yes	Yes	Yes	9	9	21
7	70	Male	25.8	3	55	Critical illness	No	No	No	Yes	0	3	11
Mean ± SD	68.1 ± 4.6	–	30.2 ± 2.3	4.2 ± 3.3	57.5 ± 16.9	–	–	–	–	–	4.7 ± 3.5	12.4 ± 7.7	21.0 ± 6.3
**Neurologic characteristics and comorbidities**													
**Patient**	**EDSS** **≤6**	**RASS (D1/D5/D8)**	**Glasgow (D1/D5/D8)**	**CAM (D1/D5/D8)**	**Oxygen therapy**	**NIV**	**Hypertension**	**Diabetes mellitus**	**Obesity**	**Dyslipidemia**	**Anxiety**	**Hypothyroidism**	**COPD**
1	Yes	−2/0/0	•/15/15	–/–/–	Yes	Yes	No	No	Yes	No	No	Yes	No
2	Yes	1/0/0	•/15/15	–/–/–	Yes	Yes	Yes	No	Yes	No	No	No	No
3	Yes	1/0/0	•/15/15	+/–/–	Yes	No	No	No	No	Yes	No	Yes	No
4	Yes	−4/0/•	•/15/•	–/–/•	Yes	No	No	No	No	No	No	No	No
5	Yes	0/0/0	15/15/15	–/–/–	Yes	No	Yes	Yes	Yes	Yes	No	No	No
6	Yes	−5/−5/•	•/•/•	–/–/•	Yes	Yes	Yes	No	Yes	Yes	No	No	No
7	Yes	0/0/0	15/15/15	–/–/–	Yes	Yes	Yes	No	No	No	No	No	Yes

*BMI, body mass index; CAM, Confusion Assessment Method; COPD, chronic obstructive pulmonary disease; EDSS, Expanded Disability Status Score; ICU, intensive care unit; IMV, invasive mechanical ventilation; NIV, non-invasive ventilation; RASS, Richmond Agitation Sedation Scale; SAPS III, Simplified Acute Physiology Score III; SOFA, Sequential Organ Failure Assessment; •, not applicable*.

### Muscle Mass Outcomes

**Figure 2:**
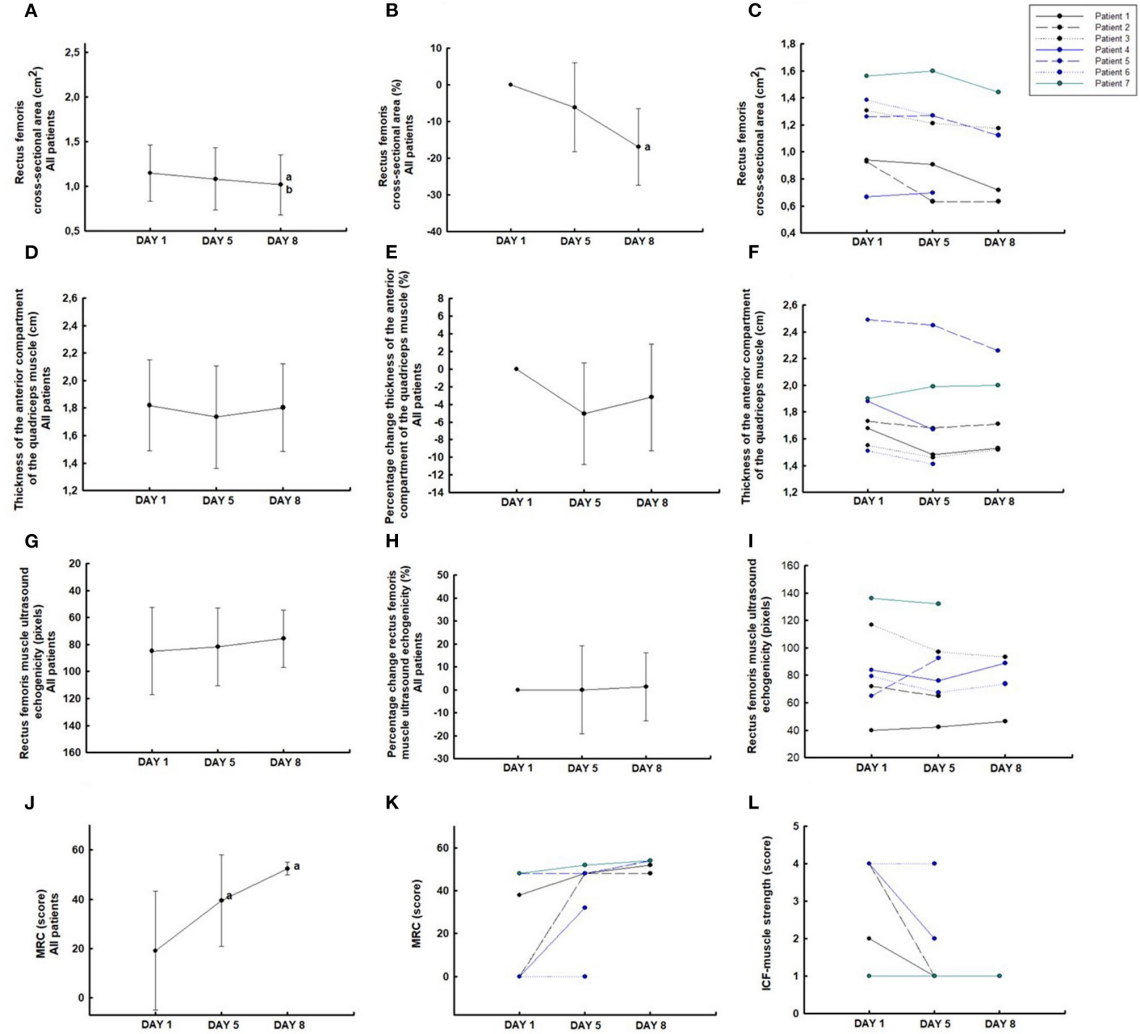
Ultrasound muscle assessement of the rectus femoris cross-section área **(A–C)**; the thickness of the anterior compartment of the quadriceps muscle (rectus femoris and vastus intermedius) **(D–F)**; rectus femoris echogenicity **(G–I)**; MRC score **(J,K)**; and International Classification of Functioning, Disability, and Health (ICF)-muscle strength **(L)**. ^a^*P* < 0.05 compared to day 1; ^b^*P* < 0.05 compared to day 5.

The rectus femoris cross-sectional area decreased significantly (−16.9% [95% CI, −29.8 to −3.9]; *P* < 0.05) from days 1 to 8 (Figures 2A–C), but showed maintenance of the thickness of the anterior compartment of the quadriceps muscle (−3.20% [95% CI, −10.6 to 4.2]; *P* = 0.3) from days 1 to 8 (Figures 2D–F). These patients showed a reduction of 2.1% [95% CI, −3.7 to −0.5] per day in the rectus femoris cross-sectional area and 0.3% [95% CI, −1.3 to 0.5] per day in the thickness of the anterior compartment of the quadriceps muscle during 8 days. Furthermore, patients showed maintenance of the echogenicity (1.3% [95% CI, −17.1 to 19.7%]; *P* = 0.8) from days 1 to 8 with an increase of 0.16% per day (Figures 2G–I).

### Peripheral Muscle Strength and Functional Outcomes

The MRC score increased significantly from days 1 to 5 (*P* < 0.05) and kept this improvement until day 8 (*P* = 0.5) (Figure 2J). In the five patients evaluated, all (100%) showed an increase in the MRC score (Figure 2K) and reduction of the ICF-muscle strength, meaning improved muscle strength from days 1 to 8 (Figure 2L). Four (80%) patients evaluated showed an increase in the MRC score and one (20%) maintained the MRC score values from days 5 to 8. Three patients (60%) showed a decrease in the ICF-muscle strength from days 1 to 5 and these values were maintained on day 8. Two patients (40%) maintained the ICF-muscle strength on days 5 and 8 compared with the baseline values (day 1).

**Figure 3:**
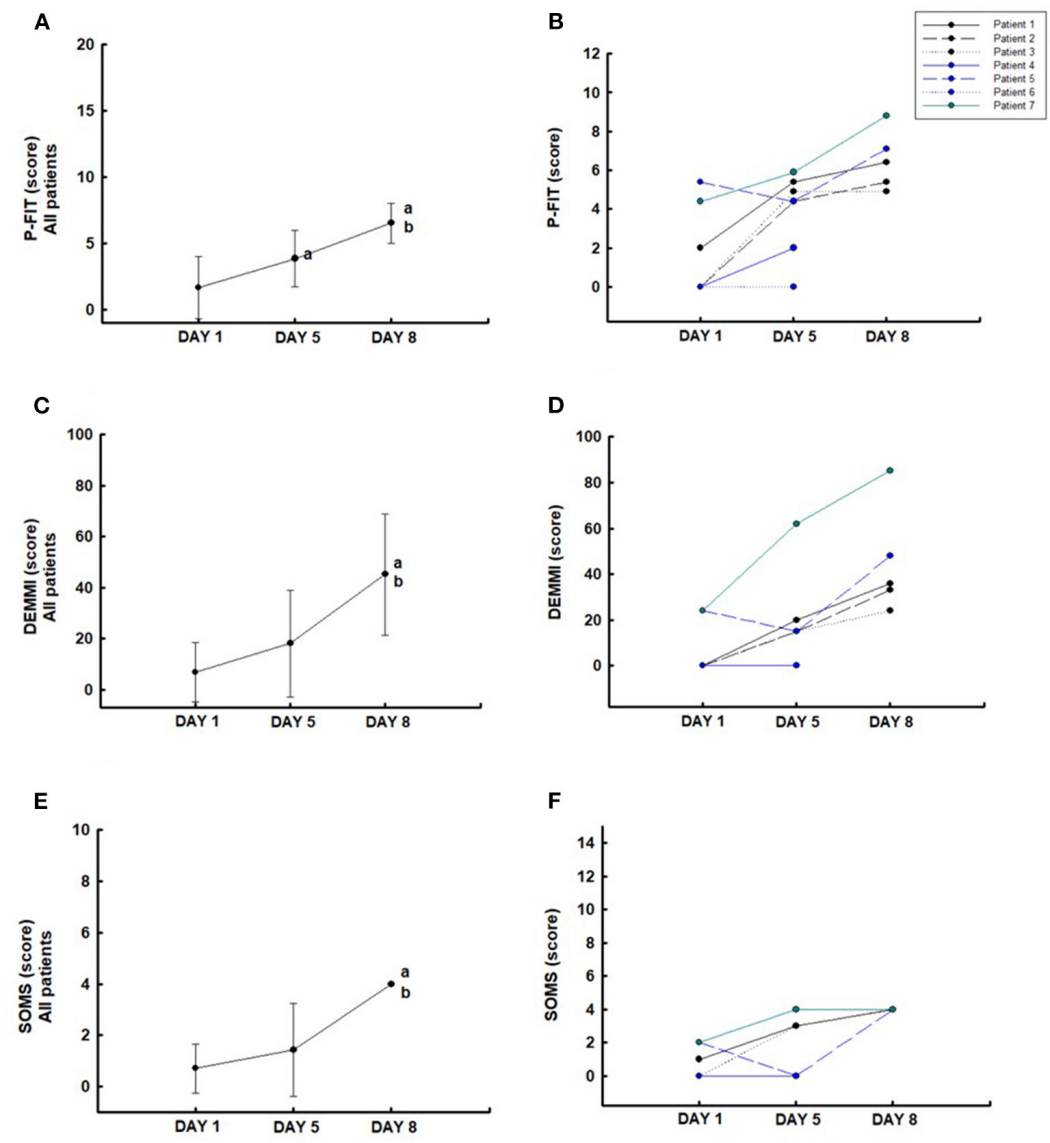
Functionality: Physical Function ICU Test-scored (PFIT-s) **(A,B)**, Morton Mobility Index (DEMMI) **(C,D)**, and the Surgical Intensive Care Unit Optimal Mobilization Score (SOMS) **(E,F)**. ^a^*P* < 0.05 compared to day 1; ^b^*P* < 0.05 compared to day 5.

The PFIT-s increased significantly from days 1 to 5 and improved until day 8 compared to day 5 (*P* < 0.05) (Figure 3A). All patients (100%) showed an increase in the PFIT-s on day 5 compared to day 1 and improvement on day 8 compared to day 5 (Figure 3B). DEMMI (Figure 3C) and SOMS (Figure 3E) scores increased significantly on day 8 compared to days 1 and 5 (*P* < 0.05). In the five patients evaluated, the individual data present that all (100%) patients showed an increase in the DEMMI (Figure 3D) and SOMS (Figure 3F) scores on days 5 and 8 compared with the baseline values (day 1).

### Feasibility and Safety

No adverse events were reported during the case series. Five patients completed the assessments and intervention. One patient interrupted the NMES intervention but did not claim intolerance during the application, and one patient died due to worsening pulmonary and respiratory conditions. None of the NMES intervention sessions were interrupted by pain.

## Discussion

Patients with COVID-19 associated with sepsis and septic shock treated with NMES presented a reduction of 16.9% in the rectus femoris cross-sectional area, but with no significant reduction in the thickness of the anterior compartment of the quadriceps muscle (3.2%) and no significant increase of rectus femoris echogenicity on day 8 (1.3%). The magnitude of these alterations was 2.1, 0.3, and 0.16% per day, respectively. We emphasize that these reported values in the present case series are smaller than those found compared to another study conducted at the same hospital and research group that evaluated severe COVID-19 patients without NMES intervention. This study showed a reduction of 30.1% in the rectus femoris cross-sectional area, 18.6% in the thickness of the anterior compartment of the quadriceps muscle, and increase of 16.8% in the echogenicity on day 10 with themagnitude of these alterations being about 3.7, 2.1, and 1.68% per day, respectively (5).

The ability of electrical muscle stimulation to improve or maintain strength, muscle mass, and functionality in ICU patients with sepsis is controversial. However, the results seem to be related to the type of stimulation frequency involved in muscle stimulation. Rodriguez et al. (9) used high stimulation frequency in the neuromuscular electrical stimulation and showed a preventive effect in the progression of muscle weakness in patients having severe sepsis requiring mechanical ventilation. On the other hand, when Poulsen et al. (8) used low stimulation frequency in the patients with septic shock admitted to the ICU, and showed that loss of muscle mass was unaffected by electrical muscle stimulation. Our results corroborate with the Rodriguez et al. (9) study and enhance the possible benefit of using high stimulation frequency for muscle electrical stimulation.

The effect of electrical muscle stimulation on muscle mass and strength can be explained by several factors. Nuhr et al. (20) and Hambrecht et al. (21) showed that NMES induces an increase in oxidative capacity with the transition from fast to slow fiber types associated with a decrease in anaerobic enzymes levels. All physiological muscle changes found with the use of electrical muscle stimulation in critically ill patients suggest that the origin is a systemic effect on microcirculation (22). Vanderthommen et al. (23) showed that in the identical levels of workload (10% of the quadriceps maximum isometric voluntary torque), the muscle reaches higher values in blood flow and oxygen consumption during NMES compared with voluntary muscle contractions. Moreover, a single session of NMES is sufficient to stimulate the increased levels of mRNA for IGF binding protein-4 (84%), MyoD (83%), myogenin (~3-fold), cyclin D1 (50%), and p21-Waf1 (16-fold), which are indicative of the initiation of myogenic processes in skeletal muscle. In the same study, an additional NMES session (a total of 14 min spread over 2 days), was sufficient to induce an increase in the concentration of total skeletal muscle ribonucleic acid (RNA) (24), most likely representing an increase in muscle protein synthesis. These results indicate that molecular adaptations of skeletal muscle to loading respond in a very short time.

Neuromuscular blocking agents cause skeletal muscle relaxation by blocking the transmission of impulses at the neuromuscular junction (25). NMES evokes a muscle contraction by activating intramuscular branches of the nerve to the muscle and not the muscle fibers directly (26) and selected brain regions in a dose-response manner (27). The use of neuromuscular blocking agents during NMES intervention may interfere with the performance of muscle contraction. However, neuromuscular blockers present a recovery time of 8–40 min after their interruption (28). Therefore, we performed NMES intervention after interrupting the neuromuscular blocking agents.

Sedation is commonly used in patients admitted to the intensive care unit (29). Dirkes et al. (30) showed that NMES represents an effective and feasible interventional strategy to prevent skeletal muscle atrophy in a fully sedated patient with critically ill. In the same study, the non-stimulated leg showed substantial type 1 and type 2 muscle fiber atrophy (a 16 ± 9 and 24 ± 7% decline in muscle fiber; respectively). In contrast, no atrophy was observed in the muscle fibers collected from the stimulated leg. Although sedation does not interfere with NMES intervention, it can compromise functional assessments. Therefore, in the present study, the MRC score, P-FITs, and DEMMI evaluations were performed only after sedation withdrawal.

The limitation of this study is that it is a single-center study design and there is no control group to compare the efficacy. In addition, the number of cases is small and it is unclear whether the results can be generalized. Mateo et al. (31) used functional electrical stimulation associated with cycling in patients post-hospitalization in the ICU for a critical form of COVID-19. However, the present case series is the first report of the effects of neuromuscular electrical stimulation intervention in patients with severe COVID-19 in the acute phase of the disease associated with sepsis and septic shock. Randomized clinical trials with more patients reporting the efficacy of electrical stimulation using NMES in patients with COVID-19 associated with sepsis and septic shock are needed to confirm our findings.

## Conclusion

Rehabilitation with NMES showed improvement in muscle strength and functionality of patients in this case series with a potential protective effect on muscle mass loss in patients with critical COVID-19 associated with sepsis and septic shock.

## Data Availability Statement

The raw data supporting the conclusions of this article will be made available by the authors, without undue reservation.

## Ethics Statement

The studies involving human participants were reviewed and approved by Ethics Committee of the Hospital Sírio-Libanês (number 3,999,139). The patients/participants provided their written informed consent to participate in this study.

## Author Contributions

SG, WC, LS, IS, and WY: study design. SG, WC, and LS: data collection. RR and WY: data analysis and draft manuscript. RR, SG, WC, LS, IS, and WY: manuscript review. All authors contributed to the article and approved the submitted version.

## Conflict of Interest

The authors declare that the research was conducted in the absence of any commercial or financial relationships that could be construed as a potential conflict of interest.

## Publisher's Note

All claims expressed in this article are solely those of the authors and do not necessarily represent those of their affiliated organizations, or those of the publisher, the editors and the reviewers. Any product that may be evaluated in this article, or claim that may be made by its manufacturer, is not guaranteed or endorsed by the publisher.
